# 25 The Association Between Pain and Sleep Post-burn Injury: A Preschool LIBRE1-5 Study

**DOI:** 10.1093/jbcr/irac012.028

**Published:** 2022-03-23

**Authors:** Kate E Surette, Silvanys L Rodríguez-Mercedes, Pengsheng Ni, Khushbu F Patel, Camerin A Rencken, Madeleine McGwin, Renata Fabia, Carrie Tully, Tina L Palmieri, Kathleen S Romanowski, Petra Warner, Frederick J J Stoddard, Jeffrey C Schneider, Lewis E Kazis, Colleen M Ryan

**Affiliations:** Shriner's Hospitals for Children - Boston, Boston, Massachusetts; Shriners Hospitals for Children - Boston, Boston, Massachusetts; Boston University School of Public Health, Boston, Massachusetts; Shriners Hospitals for Children - Boston/MGH, Boston, Massachusetts; Shriners Hospitals for Children - Boston, Seattle, Washington; Shriners Hospital for Children - Boston, Bedford, Massachusetts; Nationwide Children's Hospital, Columbus, Ohio; Children's National Hospital, Washington, District of Columbia; UC Davis and Shriners Hospitals for Children Northern California, Sacramento, California; University of California, Davis and Shriners Hospitals for California Northern California, Sacramento, California; Shriners Children's Ohio, Dayton, Ohio; Harvard Medical School, Boston, Massachusetts; , Massachusetts; Boston University School of Public Health, Spaulding Rehabilitation Hospital, Harvard Medical School, Boston, Massachusetts; Harvard Medical School, Boston, Massachusetts

## Abstract

**Introduction:**

Quality sleep is an essential part of post-injury recovery. Sleep deprivation, including that due to pain, may hinder and slow recovery progression. Therefore, it is vital to assess long-term sleep outcomes post-burn injury. This study examined the association of sleep items with the severity of pain in pediatric burn survivors one to five years of age, using Preschool LIBRE_1-5_ (Life Impact Burn Recovery Evaluation) parent-reported data.

**Methods:**

The Preschool LIBRE_1-5_ was administered to 426 parents of burn survivors during field-testing. Three sleep-specific items within the psychological domain were assessed: “My child had frightening dreams or nightmares,” “My child had trouble staying asleep,” and “My child had trouble falling asleep at night.” Each item was scored on a 5-point Likert scale from 0 = never to 4 = always. Pain was assessed on a 4-point severity scale, from 0 = not severe to 3 = very severe and dichotomized (1= not severe and 0 = mild, moderate, and very severe). For sleep items, higher scores denote better functioning. Odds ratios for each of the three items were calculated using logistic regression to measure the association between pain severity with sleep. Lower odds ratios denote that greater pain severity is associated with more sleep problems. The multiple logistic and linear regression analysis included covariates for age at time of survey, gender, race, total body surface area burned (TBSA), Hispanic (yes/no), wound dressings, and burns to critical areas (hands, face, foot). Multiple imputations were used for missing values.

**Results:**

The sample characteristics included: mean age of 3.1 + 1.4 years, mean time since burn injury of 1.2 + 1.3 years, mean TBSA of 4.2 + 7.9, 55.2% male, and 74.2% white. In the multiple logistic regression analyses, burn survivors with severe pain had significant trouble falling asleep (OR = 0.44, 95% CI [0.24, 0.81]) and staying asleep at night (OR = 0.45, 95% CI [0.24, 0.83]). No association was found with pain severity and frightening dreams or nightmares (OR = 0.8, 95% CI (0.37, 1.75). The multiple linear regression analysis showed that higher scores on pain scales were associated with poor sleep outcomes (R²= 9.5%, *p* = 0.0016). As pain scores increased, the sleep summed score decreased by 0.46 standard deviation.

**Conclusions:**

There are important associations between pain severity and sleep outcomes. Pain management and interventions for sleep improvement may lead to better outcomes in the pediatric burn population.

